# MEDICAID ADULT DAY SERVICE CENTERS STAFFING AND SERVICE DIFFERENCES BY PROFIT STATUS

**DOI:** 10.1093/geroni/igad104.2852

**Published:** 2023-12-21

**Authors:** Eleanor Batista-Malat, Kathleen Wilber

**Affiliations:** University of Southern California, Los Angeles, California, United States; University of Southern California, Los Angeles, California, United States

## Abstract

Adult day service centers (ADSC) encompass a broad range of services, yet few studies have examined staffing and service differences in for-profit and non-profit centers. Previous long-term services and supports (LTSS) research has found that for-profit nursing homes have lower levels of staffing, however, this research has not extended to ADSCs. Data from the 2018 wave of the National Post-Acute and Long-Term Care Study were used to examine differences in staff hours per patient day (HPPD) and services offered in ADSCs by profit status. Only ADSCs that accept Medicaid were included because the majority of the responding ADSCs accept Medicaid (76.8%) and Medicaid acceptance and profit status are significantly related (p<.001). Using t-tests, there was a significant difference (p=.002) in total HPPD with higher HPPD in non-profit ADSCs, with a difference of about 13 minutes. Additionally, there were significant differences in the number of transportation (p<.001) and medical (p<.001) services offered by profit status, with for-profit ADSCs offering a higher number of services, although there was no difference in the number of social/behavioral services (p=.122). These findings suggest that ADSCs that accept Medicaid may be subject to similar incentive structures that lead to lower levels of staffing in for-profit LTSS. The higher number of transportation and medical services in for-profit ADSCs that accept Medicaid also suggests different financial models for for-profit and non-profit ADSCs. Future research should more closely examine factors associated with ADSC staffing levels and service provision, and how these measures are associated with quality of care.

